# Multivariate random effects meta-analysis of diagnostic tests with multiple thresholds

**DOI:** 10.1186/1471-2288-9-73

**Published:** 2009-11-10

**Authors:** Taye H Hamza, Lidia R Arends, Hans C van Houwelingen, Theo Stijnen

**Affiliations:** 1 Department of Biostatistics, Erasmus MC - Erasmus University Medical Center, Rotterdam, the Netherlands; 2 Wadsworth Center, New York State Department of Health, Albany, NY, USA; 3 Institute of Psychology, Erasmus University Rotterdam, Rotterdam, the Netherlands; 4 Department of Medical Statistics and Bioinformatics, Leiden University Medical Center, Leiden, the Netherlands

## Abstract

**Background:**

Bivariate random effects meta-analysis of diagnostic tests is becoming a well established approach when studies present one two-by-two table or one pair of sensitivity and specificity. When studies present multiple thresholds for test positivity, usually meta-analysts reduce the data to a two-by-two table or take one threshold value at a time and apply the well developed meta-analytic approaches. However, this approach does not fully exploit the data.

**Methods:**

In this paper we generalize the bivariate random effects approach to the situation where test results are presented with *k *thresholds for test positivity, resulting in a 2 by (*k*+1) table per study. The model can be fitted with standard likelihood procedures in statistical packages such as SAS (Proc NLMIXED). We follow a multivariate random effects approach; i.e., we assume that each study estimates a study specific ROC curve that can be viewed as randomly sampled from the population of all ROC curves of such studies. In contrast to the bivariate case, where nothing can be said about the shape of study specific ROC curves without additional untestable assumptions, the multivariate model can be used to describe study specific ROC curves. The models are easily extended with study level covariates.

**Results:**

The method is illustrated using published meta-analysis data. The SAS NLMIXED syntax is given in the appendix.

**Conclusion:**

We conclude that the multivariate random effects meta-analysis approach is an appropriate and convenient framework to meta-analyse studies with multiple threshold without losing any information by dichotomizing the test results.

## Background

Meta-analysis of diagnostic accuracy studies depends on the type of data that is available from different studies. The most frequently reported measures of diagnostic test accuracy are sensitivity and specificity or a two by two table, i.e. with a single threshold value. Meta-analytic methodologies for such kind of data have been developed to summarize sensitivity and specificity separately or jointly in a fixed or random effects context, for example [1-6]. In recent years the bivariate random effects meta-analysis of diagnostic tests has become a well established approach, which can be fitted in many statistical packages [1,2]. The bivariate approach has many advantages over separate random effects meta-analysis of sensitivity and specificity and the traditional summary receiver operating characteristics (SROC) method of Littenberg and Moses [1,2,4]. Besides it is flexible to derive different outcome measures, such as overall sensitivity and/or specificity, diagnostic odds ratio and SROC curves, from the estimated parameters.

In this article we consider the situation where diagnostic results for the test under evaluation are reported in three or more categories; for example, disease severity classified as malignant, suspect or benign. One straightforward approach often followed in practice is to dichotomize the test results into two categories and apply the well developed bivariate methods separately for each of the thresholds. When data is presented for many thresholds, a ROC can be calculated per study, and meta-analytic methods have been developed to derive a SROC from them [7-9]. Poon [10] discusses a latent normal distribution model for analysing ordinal responses with applications in meta-analysis. This model can be applied to multiple threshold diagnostic meta-analysis data. However, this paper only considered fixed effect modeling. Bipat *et al *[11] discussed a multivariate random-effects approach for meta-analysis of cancer staging. They assumed the correlations between different cancer stages to be independent, i.e. SROC curves can not be derived from their model. Specifically for diagnostic accuracy studies, Dukic *et al *[12] discussed both ordinal regression and hierarchical approaches based on latent variable modeling.

The above approaches are no direct extensions of the nowadays popular bivariate meta-analysis approach for the one threshold case. The aim of this article is to generalize this approach to the situation where test results are presented using more than one threshold or in more than two categories. Not necessarily in all studies the same number of categories is presented, however, in this article we focus on the case where the number of categories is equal across studies and we discuss how our approach can accommodate studies reported with unequal number of thresholds. Our approach can be implemented in standard statistical packages. In the methods section we briefly review the bivariate random effects approach, and we introduce the multivariate approach to meta-analyse studies that report test results with more than one threshold. We illustrate the methods using published meta-analysis data and end with a discussion.

## Methods

### The bivariate random effects (BREM) approach

For the situation where each study presents one pair of sensitivity and specificity with corresponding standard errors, the bivariate meta-analysis approach [13] has become a well established method [1,2,14]. The approach preserves the two-dimensional nature of the original data taking into account the between-studies correlation of sensitivity and specificity. It can be seen as an improvement on the method of Littenberg and Moses [4], which has been the standard method to construct a SROC for more than a decade.

In this section first we will introduce the bivariate random effects model (BREM) in its standard form. Subsequently we will derive another form of the model, which starts from a model for study specific ROCs and has a different parametrization. This formulation of the model is the natural one to generalize to the case where we have two or more pairs of specificity and sensitivity per study. This formulation also sheds more light on the interpretation of SROCs, which is problematic in the case where only one pair of sensitivity and specificity is available.

For study *i*, denote *ξ*_*i *_= *logit*(1 - *specificity*_*i*_) and *η*_*i *_= *logit*(*sensitivity*_*i*_). Let *x*_1*i *_be the number of true positives, *n*_1*i *_the total number of diseased subjects, *x*_0*i *_the number of false positives and *n*_0*i *_the total number of non-diseased subjects. Then the observed sensitivity and specificity for a given study *i *are *x*_1*i*_/*n*_1*i *_and (*n*_0*i *_-*x*_0*i*_)/*n*_0*i *_respectively. Note that the underlying sensitivity and specificity tend to be negatively correlated across studies because of explicit or implicit differences in the thresholds. Therefore *ξ*_*i *_and *η*_*i *_will tend to be positively correlated.

#### Between-studies variability

The between-studies model [1] is given by:(1)

The bivariate distribution of true logit transformed sensitivities and 1-specificities can be characterized by different lines. Back transforming such a line by taking the inverse logit gives a SROC. Since there are several reasonable choices for lines characterizing a bivariate normal distribution, several types of SROCs are possible. For example, a straightforward choice would be the regression of *η *on *ξ*. However, since the roles of *ξ *(specificity) and *η *(sensitivity) are interchangeable, the regression line of *ξ *on *η *is an evenly reasonable choice. The method of Littenberg and Moses chooses the regression line of *D *= *η*-*ξ *on *S *= *η *+ *ξ*. Table 1 gives an overview of 5 different choices as distinguished by Arends *et al *[2].

**Table 1:** Different choices of summary lines as resulting from the BREM:*y *= *α *+ *βx *where y = logit(sensitivity) and x = logit(1-specificity). [[Bibr B6]]

Parameter	Type of regression line				
	
	*η *on *ξ*	*ξ *on *η*	*D *on *S*	R & G	Major Axis
*β*					

*α*				-*βξ*	

R&G denotes the SROC as resulting from the method of Rutter and Gatsonis [6]

The different SROCs can be vastly different in applications, see for instance Arends *et al *[2] and the data examples. The BREM approach as introduced by Reitsma *et al *[1] and discussed by Arends *et al *[2] does not assume anything about study specific curves. The method simply leads to an estimated underlying bivariate distribution of the true sensitivities and specificities as reported by the different studies included in the meta-analysis. This means that the chosen SROC does not necessarily correspond with the true curves of the studies. The true study specific curves might have a substantially different shape, and the SROC cannot be interpreted as a kind of average or overall ROC representative for the ROCs of the different studies. There might even be no study specific curves at all, in case the diagnostic test cannot be thought of as a continuous test. However, this does not mean that the analysis does not make sense in this case, since the existence of study specific ROC curves is not assumed by the method. In the remainder of this section we introduce a new formulation of the BREM, which starts with the study specific ROCs. This will make clear under which extra assumption the BREM describes the distribution of study specific ROCs and the calculated SROC can be considered to be a real overall SROC.

Suppose that in the (*ξ*, *η*) space the study specific ROC curves are straight lines with a common slope *β*. The lines of the different studies then only differ in level, characterised by the intercept *α*_*i *_for study *i*:(2)

We assume that the *α*_*i*_'s are normally distributed with mean  and variance . The observations consist of an estimate (, ) of one pair (*ξ*_*i*_, *η*_*i*_) per study. To be able to estimate the parameters, we have to assume a model that describes how these pairs arise across studies. A straightforward assumption is that the *ξ*_*i *_values are drawn from a normal distribution with mean  and variance . This leads to the following marginal model for (*ξ*_*i*_, *η*_*i*_):(3)

This model is just the same as (1), only with a different parametrization. However, the number of parameters is one more, which means that one of them is unidentifiable. To make the model identifiable, we need a further assumption on how the *ξ*_*i*_'s in the different studies are selected. For instance we could assume that *σ*_*αξ *_is zero. This means that the individual investigators, in selecting their *ξ*_*i *_value, are not lead by the level of their line. However it is perfectly conceivable that an investigator who happens to have a ROC that is relatively low, tends to choose a relatively high value for his *ξ*_*i *_if a high sensitivity is preferred, or just a relatively low value of *ξ*_*i *_if high specificity is preferred.

If we assume that the correlation between *α*_*i *_and *ξ*_*i *_is zero, it can be seen that *β *is given by the slope of the regression line of *η *on *ξ*. In this case the *η *on *ξ *type SROC is the true SROC in the sense that it really can be interpreted as such. In the (*ξ*, *η*) space it is just the average line over the population of studies, in the ROC space it can be interpreted as a kind of median ROC.

Another assumption could be that the correlation between *η *and *α *is zero. This means that we assume  and therefore the correlation between *α *and *ξ*_*i *_is given by *ρ*_*αξ *_= -*σ*_*α*_/(*βσ*_*ξ*_). The slope, then, is rewritten as , which is the slope of the regression of *ξ *on *η*. Thus under this assumption the *ξ *on *η *type SROC is the real one.

More general, we could assume that some linear combination *aξ *+ *bη *of *ξ *and *η *is not correlated to *α*, for some value of *a *and *b*. We have already seen that if *a *= 1 and *b *= 0, the *η *on *ξ *type SROC is the correct one. If *a *= 0 and *b *= 1, then the *ξ *on *η *type is the correct one. If we assume *a *= *b *= 1, then one can check that *β *is equal to the slope of the regression of *D *= *η*- *ξ *on *S *= *η *+ *ξ*, and the Littenberg & Moses type SROC is the correct one. One can also check that the assumption *a *= *β *and *b *= 1 leads to the Rutter & Gatsonis (R&G) type SROC. The slope of their method is the geometric average of the *η *on *ξ *and *ξ *on *η *regression line, that is, their regression line always lies in between the two curves. For a detailed discussion we refer to Arends *et al *[2]

Generally, the 5 SROCS only coincide in the very degenerate case where the between-studies variances of sensitivity and specificity are equal and the correlation is one. If only the between-studies variances of sensitivity and specificity are equal then the three methods (D on S, R&G and Major axis) lead to the same SROC curve. Note that all choices of SROC curves in Table 1, except the R&G, can possibly give an improper SROC curve that runs from (*TPR *= 1, *FPR *= 1) to (*TPR *= 0, *FPR *= 0) if one of the following situation occurred. However, the chance that this occurs in practice is very unlikely.

1. The *η *on *ξ*, *ξ *on *η *and major axis methods give an improper SROC curve if and only if sensitivity and specificity are positively correlated.

2. The *D *on *S *method give an improper SROC curve if and only if *σ*_*ξη *_< - and *σ*_*ξη *_> - or *σ*_*ξη *_> - and *σ*_*ξη *_< -. In either cases sensitivity and specificity are positively correlated.

Notice that the R&G method always yields a proper SROC because it implies that the slope parameter is always positive, being equal to the ratio of the between studies standard deviations of sensitivity and specificity. It is remarkable that the R&G SROC can be calculated from two simple univariate meta-analyses on the sensitivities and specificities separately. This is an advantage since no bivariate modeling is needed at all, but it might also question its value because apparently it does not use the possible correlation between sensitivity and specificity. We conclude that in the situation where we have only one pair of sensitivity and specificity per study a calculated SROC can only be interpreted as a real overall ROC under an untestable assumption. The assumption is especially sensitive when the differences among the estimated between-studies variances and covariance of sensitivity and specificity are large. This issue seems to have been overlooked in the literature. In a recent letter in Biostatistics Chu and Guo [15] claim that the SROC given by Harbord et al [14] and Rutter and Gatsonis [6] are "are incorrect and potentially misleading". Chu and Guo assume that the *η *on *ξ *type is the "real" SROC, but they forget that this one is based on an untestable assumption too. However, the situation changes as soon as more pairs of sensitivity and specificity are available per study.

#### Within-study variability

The within-study variability can be modeled using an approximate normal distribution [1,2] or a binomial distribution [2,14,16]. Hamza *et al *[17,18] compared in extensive simulation experiments the binomial and approximate normal within-study models, and showed that in general the performance of the binomial within-study model is much better. Chu and Cole [16] also showed similar results using a selected number of simulations. Therefore in this paper we restrict to the binomial within-study model. For the approximate approach we refer to [1,2].

The within-study model is based on the binomial distribution of the number of false positive (*x*_0*i*_) and true positive (*x*_1*i*_) test results. More specifically we assume:(4)

The *ξ*_*i *_and *η*_*i *_= *α*_*i *_+ *βξ*_*i *_are the true logit transformed (1-specificity) and sensitivity from (2). The bivariate model given by (2-4) can be fitted using generalized linear mixed model procedures in standard statistical packages, such as the SAS procedure NLMIXED, STATA *gllamm *or the R/S-Plus program *nlme*.

### Multivariate random effects meta-analysis (MREM)

In this section we consider studies where a single test is administered and the results are reported using *J *- 1 thresholds or, equivalently, with *J *ordered categories; see for example references [19,22] and table 2. Let the number of non-diseased and diseased patients with test result in category *j *from the *i*^*th *^study be given by *x*_0*ij *_and *x*_1*ij*_, respectively. The total number of non-diseased and diseased patients for study *i *is denoted by  and , respectively. For any given threshold *j *sensitivity and specificity are calculated by  and  respectively. For example, from table 2, if we consider *j *= 2, i.e. malignant and suspect as test positive, *sensitivity *= (*x*_*i*1 _+ *x*_*i*2_)/*n*_1*i *_and *specificity *= *x*_0*i*3_/*n*_0*i*_.

**Table 2:** Two-by-three contingency table for study *i *for relating the FNAC outcome to the final diagnosis of breast lesion (The FNAC data is given in the additional file 1).

FNAC outcome	Malignant	Suspect	Benign	Total
Final diagnosis				
Malignant	*x*_1*i*1_	*x*_1*i*2_	*x*_1*i*3_	*n*_1*i*_
Benign	*x*_0*i*1_	*x*_0*i*2_	*x*_0*i*3_	*n*_0*i*_

#### The model

Let the true logit transformed 1 - *specificity *and *sensitivity *for a given threshold *j *be denoted by *ξ*_*ij *_and *η*_*ij *_respectively, where *ξ*_*ij*_'s and *η*_*ij*_'s are ordered in the *j *index. We assume a hierarchical model that is a direct generalization of model (2-3). In contrast to the one threshold case (bivariate approach), when we have more than one threshold (multiple points per study), the SROC curve is identifiable. The between and within-study models are given as follows:

#### Between-studies model

1. Model for the relation between *ξ*_*ij *_and *η*_*ij*_:

Within a study we assume a linear relation with common slope *β *and study specific intercept *α*_*i*_.(5)

The parameters  and *β *determine the summary ROC curve. Note that *β *is an asymmetry parameter. If *β *= 1 then the curve is symmetric around the line of equal sensitivity and specificity. We could also allow *β *to vary across studies and assume a bivariate normal distribution for the pair *α*_*i *_and *β*_*i*_.

2. Model for the *ξ*_*ij*_'s:(6)

Here  is the mean *ξ*_*ij *_over studies and the 's are constrained to keep their rank order, Δ_*i *_represents the study specific systematic deviation of the *ξ*_*ij*_'s from the overall means  and *δ*_*ij *_represents the random residual deviation of true unobserved observations. The Δ_*i*_'s can be assumed to follow some parametric or non-parametric distribution. In this article we assume a normal distribution given by Δ_*i*_*~N *(0, ). The *δ*_*ij*_'s are assumed to be independent and follow a normal distribution, *δ*_*ij*_*~N *(0, ). Furthermore, the *δ*_*ij*_'s are assumed to be independent of the Δ_*i *_and *α*_*i*_. The covariance between *α*_*i *_and Δ_*i *_is denoted by *σ*_*α*Δ_. A negative *σ*_*α*Δ _for instance would mean that in studies with a relatively small *α*_*i *_the *ξ*_*ij*_'s tend to be chosen relatively high. The above assumptions (in 5 & 6) lead to the following marginal between-studies model:(7)

Note that the covariance structure for the *ξ*_*ij*_'s is of compound symmetry, i.e. the between-studies variances of *ξ*_*ij*_'s are assumed to be equal and the covariances between any of the *ξ*_*ij*_'s are assumed to be the same. We have chosen this structure since it is popular in repeated measures modeling as a simple but often realistic covariance structure. Moreover the parameters have a nice interpretation. However, one can choose any structure. The assumption of compound symmetry might be strong; for example, the covariance (correlation) between consecutive thresholds may be larger compared to between non-consecutive ones. If so, a richer structure is needed. In general, the between-studies variances and covariances of *ξ*_*ij*_'s could be allowed to be all different (unstructured covariance matrix) and, in the spirit of the general guidelines for mixed model building given by Verbeeke and Molenberghs [23](chapter 9), it could then be simplified into another covariance structure, such as Toeplitz, auto-regressive or compound symmetry.

#### Within-study model

Given (*α*_*i*_, *β *, *ξ*_*i*1_, ⋯, *ξ*_*i*, *J*-1_), the observed number of subjects in the non-diseased (*x*_0*i*1_, ⋯, *x*_0*iJ*_) and diseased (*x*_1*i*1_, ⋯, *x*_1*iJ*_) groups have independent multinomial distributions with parameters (*π*_0*i*1_, ⋯, *π*_0*iJ*_) and (*π*_1*i*1_, ⋯, *π*_1*iJ*_), where(8)

Note that *ξ*_*ij *_is logit(1-specificity) and *η*_*ij *_is logit(sensitivity) for threshold *j*, and the cell probabilities for diseased (or non-diseased) subjects for category *j *is the difference of sensitivities (or 1-specificities) between category *j *and *j *- 1.

The probability density function (pdf) given the *π*_0*ij*_'s and *π*_1*ij*_'s of the observations of the *i*^*th *^study is given by:(10)

Inference on the parameters is obtained through the standard likelihood method based on the marginal density for the data, which is calculated by integrating out the random effects **B **= (*α*, *ξ*_1_, ⋯, *ξ*_*J*-1_)'. Then the contribution of the *i*^*th *^study to the likelihood is(11)

As seen from 7, we assumed a multivariate normal distribution for the random effects. However, the density *g*(**B**) can also be assumed to belong to some other parametric family of distributions [24]. In summary the MREM model allows to calculate different relevant measures of diagnostic test accuracies:

• The mean logit sensitivity  and logit specificity  along with their standard errors for any known threshold, *j*, are estimated by the model. One can then derive sensitivity and specificity as follows: *sensitivty*_*j *_=  and *specificity*_*j *_= , and the corresponding standard errors can be calculated using delta method [25]. SAS NLMIXED users avoid hand calculation by using the 'Estimate' statement (see the SAS syntax for example).

• The diagnostic odds ratio (DOR), that can be derived from sensitivity and specificity, for a given threshold, *j*, is given by *DOR*_*j *_= 

• The estimated parameters from model (5 - 7) are used to derive the overall median SROC curve that is given by *sensitivity*(*specificity*) = . Besides, study specific ROC curves can be generated from the empirical Bayes estimates of the random effects model. In SAS NLMIXED, the empirical Bayes estimates are generated automatically and collected from the output file ('out') specified in the 'random' statement (see SAS syntax).

• Uncertainty around the SROC can be characterized by calculating the confidence interval at each point along the curve.

• A prediction band for the true ROC curve of a new study can be calculated by adding and subtracting 1.96 times the estimated standard deviation of *α*_*i *_in the equation above.

• The model accommodates study level covariates, for example corresponding to two different diagnostic tests, that enables to test the hypothesis for the differences between groups, e.g. diagnostic tests. A thorough discussion of comparison between groups or test results using the bivariate model is given in Hamza *et al *[26]. In the second data example of this paper we show how to test for the significant difference between groups.

#### Fitting the model

The parameters of interest from the MREM approach (, *β*, 's along with their between-studies variances and covariances , *σ*_*α*Δ_, , ,) that are used to calculate different diagnostic test accuracy measures can be estimated as follows.

1. We fitted the model using Proc NLMIXED of SAS. This procedure does not support directly the multinomial distribution. However, the procedure allows a user specified log-likelihood function. This is easily done for the multinomial distribution. In the appendix the syntax for an example is given. The NLMIXED procedure calculates the likelihood function by numerical integration, using adaptive Gaussian quadrature. The number of quadrature points is specified by the user or automatically by SAS. The larger that number is chosen the better the approximation, but at the cost of more computational time. For a detailed discussion of different choices of quadrature options, optimization methods and convergence criteria we refer to the SAS manual [27]. In practice, when a final model is reached, one increases the number of quadrature points until the parameter estimates do not change anymore.

2. NLMIXED allows user specified likelihoods, but many other programs do not. Usually the binomial distribution is supported, therefore we also mention another possibility to fit the model in Generalized Linear Mixed Model programs. The trick is to write the multinomial pdf as a sequence of conditional univariate pdf's, i.e. the pdf of (*x*_*Di*1_, ⋯, *x*_*DiJ*_) is expressed as:

where *D *is the disease status, 0 or 1. These conditional distributions are all binomial [28] and given by:(12)

where *π*_0*ij *_and *π*_1*ij *_are calculated as in (8 & 9) with *j *= 1, ..., *J *- 1.

## Results

To illustrate the methods discussed in this article, we apply them to two published meta-analysis data-sets. One is relatively large (29 studies) with three test result categories (2 thresholds). The second data set is small (10 studies) with five test result categories (4 thresholds). Here our objective is to fit the models discussed in the methods section, and to derive the SROC curves.

### Example 1: Fine-needle aspiration cytologic examination

Giard and Hermans [19] present 29 studies evaluating the accuracy of fine-needle aspiration cytologic examination (FNAC) of the breast to assess the presence of breast cancer (see additional file 1. FNAC provides a non-operative way of obtaining cells for the establishment of the nature of a breast lump and therefore plays a pivotal role in the preoperative diagnostic process [19,20]. The selected FNAC results were classified in the following four cytologic categories: definitely malignant, suspect for malignancy, benign, and unsatisfactory specimen for diagnosis (acellular aspiration). The last category is for those who do not have satisfactory specimen for diagnosis. Following the authors, we merged this group with the benign group, which resulted in a two by three table (Table 2).

**Figure 1:**
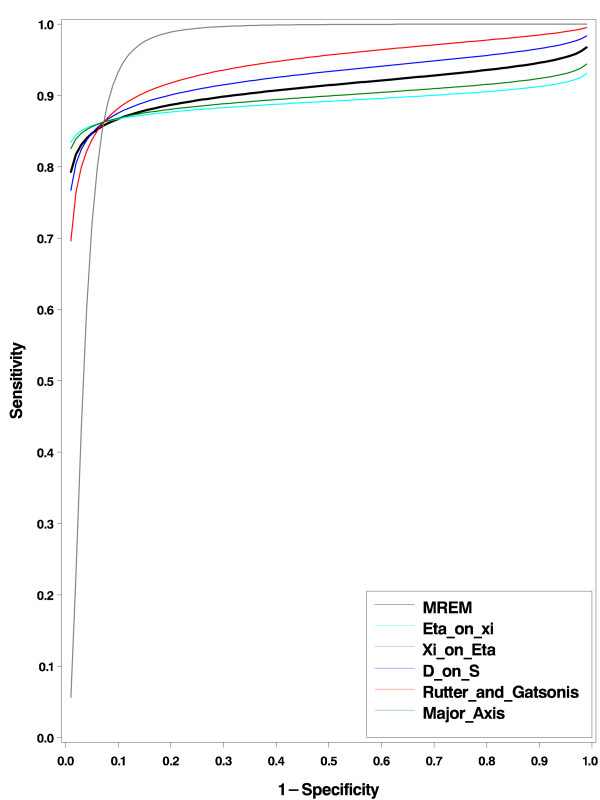
**SROC curves from the five choices of BREM approach (red = R&G, blue = D-on-S, green = Major axis, cyan = *η*-on-*ξ *and gray = *ξ*-on-*η*) and MREM approach (black) for the FNAC data set**.

The authors [19] determined the sensitivity and specificity of FNAC for each study by reducing the two-by-three table into a two-by-two table. They classified malignant and suspect test results as test result positive, and benign as test result negative. Here, following this classification, we applied first the BREM introduced in the method section. The estimated median specificity and sensitivity (standard error) are 0.927(.0015) and 0.863(0.014) respectively, and the covariance parameters are estimated as  = 1.306(0.416), *σ*_*ξη *_= 0.139(0.156) and  = 0.317(0.105). From these estimates, the 5 different types of SROCs were calculated and depicted in figure 1. The corresponding intercepts and slopes, and area under the curve (AUC) are given in table 3. Notice that there are relatively large differences between these curves. As argued in the BREM method section, from the BREM the true SROC is not identifiable. The different curves correspond with assuming a correlation of *α*_*i *_and *ξ*_*ij *_(*ρ*_*αξ*_) equal to 0.000, -0.976, -0.400, -0.620 and -0.085 respectively for types 1 to 5 mentioned in table 1. Note that when the absolute correlation increases the test accuracy as expressed by the AUC increases. However the choice of the correlation remains questionable and one should be careful because it is not identifiable from the data without extra assumptions.

**Table 3:** Parameter estimates (standard errors) and AUC of the SROC curves from the BREM and MREM approaches for the FNAC data

Type of SROC	*α*	*β*	AUC
BREM			
*η *on *ξ*	2.110(0.321)	0.107(0.118)	0.882
*ξ *on *η*	7.636(6.307)	2.276(2.463)	0.955
*D *on *S*	2.643(0.371)	0.316(0.137)	0.918
Rutter and Gatsonis	3.094(0.319)	0.493(0.112)	0.935
Major axis	2.191(0.406)	0.138(0.153)	0.889

MREM	2.368(0.135)	0.224(0.016)	0.902

Next we fitted the MREM method. The estimated median specificities (standard error) at the first and second thresholds respectively are 0.999(0.0003) and 0.928(0.018), and the corresponding sensitivities can be estimated by  and are 0.687(0.028) and 0.858(0.016). Note that the estimated median specificity and sensitivity at the second threshold are approximately equal to the BREM estimates. The estimated variances and covariances were  = 0.363(0.117), *σ*_*α*Δ _= -0.045(0.143), σ_Δ _= -0.042(0.443) and  = 1.841(0.615). The test for the significance of the covariance between *α*_*i *_and *ξ*_*i *_in the MREM approach is not significant (likelihood ratio  = 0.999, p-value = 0.317), and hence there is no indication for the choice of specificities to depend on the level of individual curves. The estimates for the intercept and slope, and AUC are given in table 3. The SROC from the MREM is depicted in figure 1. Comparing the estimated SROC curves from the two approaches, one can see that the BREM approach underestimates or overestimates the SROC curves depending on the choice of the type of SROC. This can be seen clearly from the AUC of the SROC curves in table 3. Of course, if we had chosen another cut point for the BREM, we would have ended up with a different SROC estimate for each of the choices. By the delta method it is possible to construct a confidence band for the SROC curve, as explained above. We did not do here, to avoid a too busy picture.

**Figure 2:**
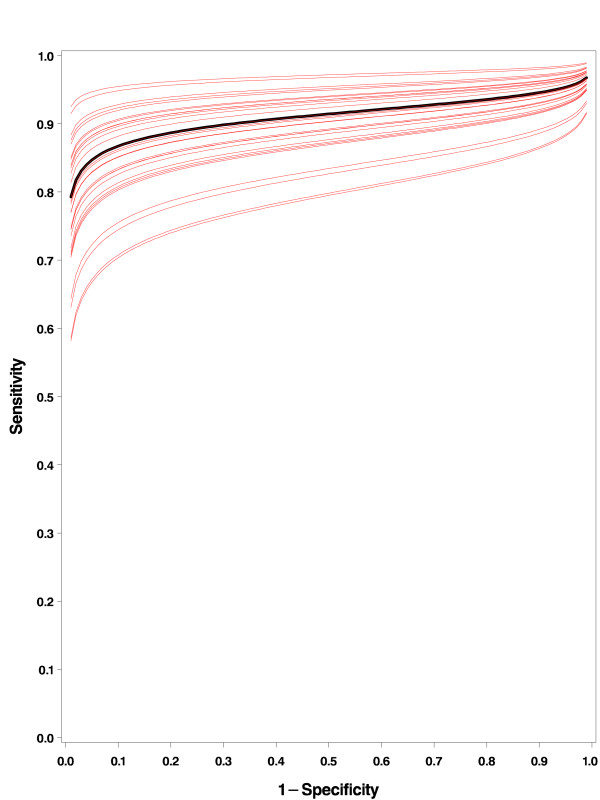
**SROC curve (black line) with 29 study specific curves (red lines) from the MREM approach for the FNAC data set**.

In contrast to the BREM, the MREM can provide estimates of the study specific ROCs. The program that we used, NLMIXED from SAS, gives the empirical Bayes estimates of the study specific random intercept *α*_*i*_, which enables to draw study specific SROC curves. We give the study specific ROCs from the MREM approach in figure 2. Indeed, the BREM can also provide study specific curves, but only if an untestable assumption on the correlation between *α*_*i *_and *ξ*_*i *_is made. Note that the study specific curves in figure 2 are "parallel". This is because we assumed a common fixed slope parameter *β *across studies. Allowing *β *to be random might give study specific curves that might cross. In principle one can do that. Then 3 extra parameters have to be estimated: the variance of *β *and its covariance with *α *and Δ. There are enough degrees of freedom and the model can still be fitted in NLMIXED. However, the more covariance parameters, the larger the chance of non-convergence problems. When we tried to take *β *random in this example, we were not able to get the program converging, probably due to correlations becoming -1 or 1, a phenomenon already well known in the much simpler BREM [21].

### Example 2: CAGE in screening for alcoholism

**Figure 3:**
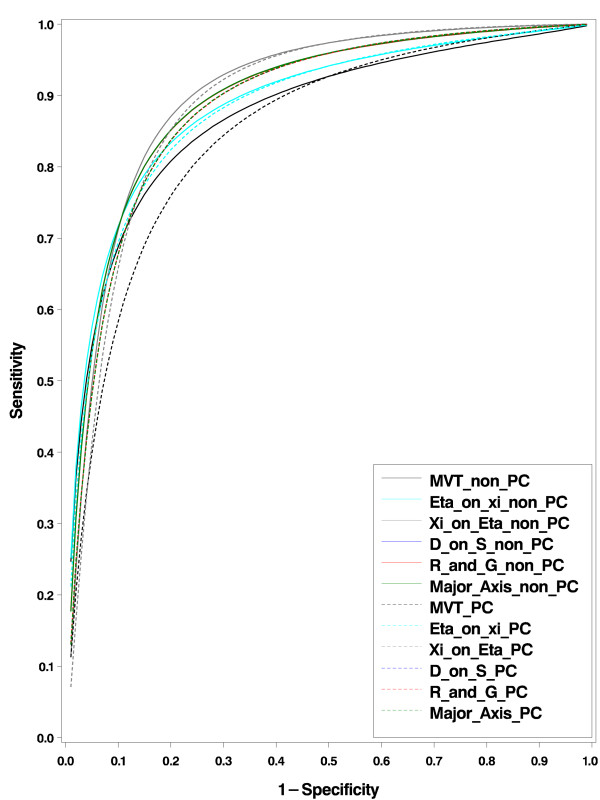
**SROC curves from the five choices of BREM approach (red = R&G, blue = D-on-S, green = Major axis, cyan = *η*-on-*ξ *and gray = *ξ*-on-*η*) and MREM approach (black) for the CAGE data set**. The curves for the primary and non-primary carry population are given in the same color but with solid and broken lines respectively. The three approaches (R&G, D on S and Major axis) give the same estimates (overlapped curves).

The CAGE questionnaire is a combination of four questions (resulting in a score from 0 to 4) that can be used for the screening of patients on alcoholism or alcohol dependence. Aertgeerts *et al *[22] performed a meta-analysis of all published studies to evaluate the diagnostic value of the CAGE questionnaire. In total they presented 10 studies published between January 1974 to December 2001, of which 5 were carried out in primary care populations and 5 in non-primary. In this data example we also include the study level covariate whether or not the patients are from primary care. If a study is carried out in a primary care population then *z*_1 _is assigned 1 else *z*_1 _is 0. Besides, for the MREM approach, the slope parameter is allowed to be random and it is tested if its between-studies variance is significantly different from zero. In most cases a CAGE score of ≥ 2 is considered to indicate an alcohol problem. For the illustration of the BREM method we therefore use the threshold of ≥ 2 as test positive. Now the mean structure in (1) or (3) is replaced by  = *a*_0 _+ *a*_1_*z*_1 _and  = *b*_0 _+ *b*_1_*z*_1 _[26]. The summary lines in the logit-logit space are then *y *= *α *+ *γ z*_1 _+ *βxb *with *α *= *b*_0 _- *a*_0_*β*, *γ *= (*b*_1 _- *a*_1_*β*)*z*_1 _and *β *is given by the different choices given in table 1. The estimated parameters (standard error) are *a*_0 _= -2.135(0.390), *a*_1 _= - 0.160(0.547), *b*_0 _= 0.982(0.393) and *b*_1 _= -0.084(0.552). The estimated specificities for non-primary and primary care patients respectively are 0.894(0.037) and 0.908(0.032), and the corresponding sensitivities are 0.728(0.078) and 0.711(0.080). The covariance parameters were estimated as  = 0.647(0.344), *σ*_*ξη *_= 0.543(0.302) and  = 0.671(0.363). The resulting estimates of the 5 lines are given in table 4 and the corresponding SROCs are depicted in figure 3. Unlike the FNAC data example, the differences between the SROC curves are small.

**Table 4:** Parameter estimates (standard errors) and AUC (for non-primary care patients (non-PC), and for primary care patients (PC)) of the SROC curves from the BREM and MREM approaches for the CAGE data-set

Type of SROC	*α*	*β*	*γ*	*AUC*_*non-PC*_	*AUC*_*PC*_
BREM					
*η *on *ξ*	2.775(0.712)	0.840(0.316)	0.050(0.389)	0.886	0.890
*ξ *on *η*	3.618(0.827)	1.235(0.364)	0.113(0.479)	0.902	0.908
*D *on *S*	3.160(0.710)	1.020(0.314)	0.079(0.419)	0.895	0.900
R & G	3.156(0.657)	1.019(0.286)	0.079(0.417)	0.895	0.900
Major Axis	3.165(0.776)	1.023(0.347)	0.079(0.420)	0.895	0.900

MREM	2.537(0.312)	0.795(0.047)	0.207(0.382)	0.849	0.888

The two-by-five tables from the CAGE meta-analysis were also analyzed using the MREM approach. Here the between-studies model in (5) can be rewritten as *η*_*ij *_= *α*_*i *_+ *β*_*i*_*ξ*_*ij *_+ *γz*_1 _to adjust for the covariate *z*_1_. Note that the slope parameter is random and assumed to be independent of *α*_*i *_and *ξ*_*ij*_; i.e. *β*_*i*_*~N *(, ). In fact the *ξ*_*ij *_can also be adjusted for study level covariates or the interaction between *ξ*_*ij *_and *z*'s could be added, but we did not do that in this example. First we tested the random slope assumption ( ≠ 0) with the likelihood ratio test using mixture of chi-square test statistics. It turned out the null hypothesis that the slope parameter is not random was not rejected (p = 0.19), and therefore we fitted the MREM model assuming fixed slope parameter. The estimated parameter for primary versus non-primary population is 0.207(0.382), P = 0.589. Therefore there is no significant difference between the two group of population. The estimated median specificities (standard error) at thresholds 1-4 respectively are 0.994(0.002), 0.976(0.006), 0.902(0.021) and 0.742(0.045). And the estimated median sensitivities (standard error) are 0.172(0.041), 0.403(0.068), 0.684(0.061) and 0.846(0.038) for non-primary care patients; and 0.203(0.046), 0.453(0.069), 0.727(0.056) and 0.871(0.033) for primary care patients. The estimated variances and covariances are  = 0.392(0.211), *σ*_*α*Δ _= -0.217(0.178), *σ*_Δ _= 0.463(0.226) and  = 0.036(0.022). The test for the correlation between the random intercept, *α*_*i *_and *ξ*_*i *_is not significant (*χ*^2 ^= 2.1, p-value = 0.147). Therefore there is no indication for the choice of the *ξ*_*i*_'s to depend on the level of individual curves. The estimated SROC parameters are given in table 4.

As shown in figure 3 and the AUCs from table 4 the bivariate approach seems to over estimate the SROC curve, for any of the 5 choices of the type of the SROC. Again this would possibly be changed if we choose another cut-off point for positivity on the screening test for alcoholism.

## Discussion

The summary ROC curve has been introduced as a way to assess the diagnostic accuracy of a diagnostic test in a meta-analysis [4,5,7,29,30]. For the most frequent situation, when one point per study is presented, the medical (and statistical) articles seem to have overlooked the problems inherent to SROCs based on studies with only one point. Although recent developments in the area have shown that the bivariate random effects meta-analysis approach has important advantages over the standard SROC approach of Littenberg and Moses [1,2,4], the problem of identifiability and therefore interpretability of the resulting SROC remains. When studies present more than one point per study, commonly the test results are reduced to two categories and meta-analysed using a well established approach such as the BREM, which is a suboptimal approach. In our data examples we illustrated this by considering a single cut-off value and applying the BREM approach. The results from the two data examples showed that differences between the estimated SROC curves based on the BREM approach can be large, as in the first example, or relatively small, as in the second example. The sizes of the differences depend on the values of the three covariance parameters. The *η *on *ξ *and *ξ *on *η *curves are always most extreme in the sense that the other three lie between them. Therefore a necessary and sufficient condition for the 5 different curves to be equal is the correlation being one, which is not very probable in practical situations. Equality of the variances of *ξ*_*i *_and *η*_*i *_is a sufficient condition for equality of the SROCs from the three intermediate approaches (D on S, Rutter and Gatsonis, and major axis). In the first example the variances differ a factor 4 and the correlation is relatively small (0.22). In the second example the variances are almost equal and the correlation is relatively large (0.82). This explains why the differences are large in the first and small in the second example.

In this article we generalized the BREM approach for one threshold to the situation where more than one point per study is available and the number of thresholds is equal across studies. In our opinion, the MREM approach is relatively easy to understand and has several advantages. First, data of the full 2 by *k *table is used without losing any information by dichotomizing the test results. Second, different outcome measures can be derived from the fitted model, such as SROC curves and overall sensitivity and/or specificity for any choice of the threshold. Third, in contrast to the BREM approach, the summary ROC and the study specific curves are identifiable. Fourth, the model is symmetric in the *ξ*_*ij*_'s and *η*_*ij*_'s. Interchanging their role leads to the same model. Fifth, it is straightforward to include study level covariates. They can be added directly to the intercept and slope of the SROC, and also to the threshold values. Sixth, the MREM can be fitted in standard statistical packages without extra programming. In equation (7), we specified compound symmetry for the covariance structure of the *ξ*_*ij*_'s. However, one can also choose another, possibly richer structure and simplify it using the likelihood ratio test. However, more covariance parameters gives more risk of non-convergence problems. For the within-study model we used the multinomial distribution instead of the summary statistic approach usually followed in meta-analysis. This avoids problems with small numbers and zero cells are allowed.

We used NLMIXED from SAS to fit our models and noticed that convergence of the program is sensitive for starting values. It turned out that good starting values are obtained by fitting the BREMs according to all possible cut-off values.

Related work was done by Dukic and Gatsonis [12]. They used ordinal regression and a hierarchical approach based on latent variable modeling and fitted their model by Bayesian methods. To our knowledge their approach is rarely used in practice, probably due to the inherent complexity of the model and fitting methods. The difference between Dukic and Gatsonis model and the MREM is mainly in the modeling of the *ξ*_*ij*_'s. They treated them all as fixed parameters, leading to as many *ξ *parameters as there are data points, while we modeled them using the standard multivariate meta-analysis model [13]. The motivation for this is to reduce the number of parameters and to correct for the measurement errors in the 's. In our opinion, Dukic's method does not correct for measurement error in the 's and leads to an inconsistent estimate of the summary ROC curve, for reasons set out in Van Houwelingen and Senn [31]. Another difference is that Dukic's method assumes independence between the choice of the specificities as represented by the *ξ*_*ij *_parameters and the level of the study specific ROC curve as determined by *α*_*i*_; a not necessarily realistic assumption.

### Model Extension and Limitation

In this paper we focused on the situation where test results are presented with equal numbers of thresholds, but the method is more general. In practice test results can possibly be presented with different numbers of thresholds. Often the different numbers of categories arises while all studies in principle use the same categorization, but in different studies different categories are lumped together. Then our model can still be applied without any modification, since our approach allows missing data points. Many other situations can be covered by allowing the parameters of the model for the *ξ*_*ij*_'s (6) to be different for different groups of studies. For instance, suppose the goal of the meta-analysis is to compare two tests A and B, one with 3 and the other with 5 categories, and each study reporting either A or B. Then the *ξ*_*ij *_model for A can be specified to be completely different from that of B. Such a model can still be estimated in for instance using Proc NLMIXED. The total number of different thresholds across all studies is the limiting factor in our approach. If it is too large, the number of parameters might be too large to estimate and the likelihood method may not work properly.

## Conclusion

The multivariate random effects meta-analysis approach is an appropriate and convenient framework to meta-analyse studies with multiple threshold without losing any information by dichotomizing the test results. The model can be implemented on standard statistical packages.

## Abbreviations

ROC: Receiver operating characteristics; SROC: Summary receiver operating characteristics; BREM: Bivariate random effects model; MREM: Multivariate random effects meta-analysis; FNAC: Fine-needle aspiration cytologic examination; AUC: Area under the curve.

## Competing interests

The authors declare that they have no competing interests.

## Authors' contributions

THH, LRA, HCvH, TS developed the study. THH performed the analysis of FNAC and CAGE supervised by TS. THH wrote the paper with contributions from LRA, HCvH and TS. HCvH and TS supervised the study. All authors read and approved the final manuscript.

## Appendix

SAS syntax to fit the MREM model by writing the likelihood in the SAS procedure NLMIXED

*PROC NLMIXED = call SAS procedure NLMIXED, and the options included represents:

   df = degree of freedom, we specified 1000 to get Wald test in stead of t-test

   qpoints = number of quadrature points, if not specified SAS automatically does

   miniter = is the minimum number of iteration;

PROC NLMIXED DATA = cage DF = 1000 MINITER = 30 QPOINTS = 5;

*Specifies initial values for the maximum likelihood estimates

   if not specified, all the initial values automatically assigned to be one;

PARMS ma = 2.6 b = 0.8 b2 = 0 mxi1 = -5.2 mxi2 = -3.7 mxi3 = -2.2 mxi4 = -1.0

         va = 0.4 cavdi = -0.2 vdi = 0.5 vdij = 0.02;

*Constraint to make sure that xi's are in the right order;

      BOUNDS mxi1-mxi2< = 0, mxi2-mxi3< = 0, mxi3-mxi4< = 0;

*Between-studies model (equation (5));

      eta1 = a + b*xi1 + b2*z1;

      eta2 = a + b*xi2 + b2*z1;

      eta3 = a + b*xi3 + b2*z1;

      eta4 = a + b*xi4 + b2*z1;

*Within-study model (equation (8));

      p01 = 1/(1+exp(-(xi1)));

      p02 = 1/(1+exp(-(xi2))) - 1/(1+exp(-(xi1)));

      p03 = 1/(1+exp(-(xi3))) - 1/(1+exp(-(xi2)));

      p04 = 1/(1+exp(-(xi4))) - 1/(1+exp(-(xi3)));

      p05 = 1 - 1/(1+exp(-(xi4)));

*Within-study model (equation (9));

      p11 = 1/(1+exp(-(eta1)));

      p12 = 1/(1+exp(-(eta2))) - 1/(1+exp(-(eta1)));

      p13 = 1/(1+exp(-(eta3))) - 1/(1+exp(-(eta2)));

      p14 = 1/(1+exp(-(eta4))) - 1/(1+exp(-(eta3)));

      p15 = 1 - 1/(1+exp(-(eta4)));

*Log-likelihood (equation (10));

   if (p01^ = 0 and p02^ = 0 and p03^ = 0 and p04^ = 0 and p05^ = 0 and

      p11^ = 0 and p12^ = 0 and p13^ = 0 and p14^ = 0 and p15^ = 0) then

   ll = n01*log(p01)+n02*log(p02)+n03*log(p03)+n04*log(p04)+n05*log(p05)+

      n11*log(p11)+n12*log(p12)+n13*log(p13)+n14*log(p14)+n15*log(p15);

   else ll = -1**100;

*SAS maximized the likelihood using the general distribution;

   MODEL n11 ~general(ll);

*Random effects parameters alpha_i and xi_ij's are normally distributed around their mean and between-studies variance covariance matrix (equation 7);

   RANDOM a xi1 xi2 xi3 xi4 ~normal([ma, mxi1, mxi2, mxi3, mxi4],

            [va,

            cavdi, vdi+vdij,

            cavdi, vdi, vdi+vdij,

            cavdi, vdi, vdi, vdi+vdij,

            cavdi, vdi, vdi, vdi, vdi+vdij])

   SUBJECT = study OUT = study_specific; *Empirical Bayes estimate;

*Estimate sensitivities and specificities at each of known thresholds (1,2,3,4);

   ESTIMATE 'sensitivity_1' exp(ma + mxi1)/(1 + exp(ma + mxi1));

   ESTIMATE 'sensitivity_2' exp(ma + mxi2)/(1 + exp(ma + mxi2));

   ESTIMATE 'sensitivity_3' exp(ma + mxi3)/(1 + exp(ma + mxi3));

   ESTIMATE 'sensitivity_4' exp(ma + mxi4)/(1 + exp(ma + mxi4));

   ESTIMATE 'specificity_1' exp(1)/(1 + exp(mxi1));

   ESTIMATE 'specificity_2' exp(1)/(1 + exp(mxi2));

   ESTIMATE 'specificity_3' exp(1)/(1 + exp(mxi3));

   ESTIMATE 'specificity_4' exp(1)/(1 + exp(mxi4));

run;

## Pre-publication history

The pre-publication history for this paper can be accessed here:

http://www.biomedcentral.com/1471-2288/9/73/prepub

## Supplementary Material

Additional file 1 **Data from clinical studies on patients with a breast mass who underwent a fine-needle aspiration cytologic examination (FNAC)**. FNAC data example used in the first example to illustrate the methods discussed in the article. Click here for file
