# Supplementary material for: Multivariate random effects meta-analysis of diagnostic tests with multiple thresholds
Source: BMC Med Res Methodol. 2009 Nov 10;9:73. doi: 10.1186/1471-2288-9-73 (PMC2787531; doi:10.1186/1471-2288-9-73)
Supplement: Additional file 1 — Data from clinical studies on patients with a breast mass who underwent a fine-needle aspiration cytologic examination (FNAC). FNAC data example used in the first example to illustrate the methods discussed in the article. [file 1471-2288-9-73-S1.doc]

Data from clinical studies on patients with a breast mass who underwent a fine-needle aspiration cytologic examination (FNAC).

| Author | year | FNAC result given breast cancer | | | | FNAC result given benign disease | | | |
| --- | --- | --- | --- | --- | --- | --- | --- | --- | --- |
|  |  | Malignant | Suspect | Benign | Unsat. | Malignant | Suspect | Benign | Unsat. |
| Linsk | 1972 | 823 | 156 | 56 | 33 | 1 | 69 | 805 | 134 |
| Furnival | 1975 | 51 | 0 | 5 | 17 | 2 | 1 | 121 | 42 |
| Zajdela | 1975 | 1526 | 43 | 63 | 89 | 3 | 52 | 846 | 48 |
| Wilson | 1978 | 19 | 16 | 9 | 6 | 2 | 23 | 164 | 95 |
| Thomas | 1978 | 49 | 10 | 4 | 8 | 0 | 4 | 92 | 29 |
| Duguid | 1979 | 50 | 6 | 2 | 2 | 0 | 18 | 181 | 35 |
| Kline | 1979 | 240 | 89 | 35 | 4 | 0 | 602 | 2810 | 307 |
| Gardecki | 1980 | 109 | 16 | 6 | 11 | 0 | 10 | 146 | 67 |
| Strawbridge | 1981 | 141 | 70 | 24 | 39 | 3 | 85 | 326 | 173 |
| Shabot | 1982 | 46 | 3 | 0 | 1 | 0 | 0 | 29 | 2 |
| Azzarelli | 1983 | 262 | 74 | 65 | 113 | 3 | 23 | 381 | 262 |
| Bell | 1983 | 119 | 91 | 27 | 15 | 0 | 147 | 615 | 131 |
| Norton | 1984 | 8 | 8 | 1 | 2 | 0 | 5 | 9 | 16 |
| Dixon | 1984 | 222 | 36 | 24 | 29 | 0 | 16 | 275 | 81 |
| Aretz | 1984 | 26 | 30 | 14 | 4 | 0 | 9 | 93 | 14 |
| Ulanow | 1984 | 137 | 25 | 19 | 9 | 1 | 15 | 100 | 12 |
| Wanebo | 1984 | 93 | 23 | 1 | 12 | 0 | 6 | 102 | 10 |
| Wollenberg | 1985 | 52 | 13 | 11 | 1 | 0 | 99 | 132 | 13 |
| Somers | 1985 | 81 | 13 | 5 | 5 | 0 | 5 | 37 | 41 |
| Lannin | 1986 | 23 | 3 | 2 | 2 | 0 | 0 | 63 | 7 |
| Eisenberg | 1986 | 1050 | 268 | 72 | 177 | 0 | 28 | 68 | 68 |
| Barrows | 1986 | 481 | 88 | 48 | 72 | 2 | 53 | 338 | 201 |
| Watson | 1987 | 37 | 9 | 13 | 3 | 1 | 0 | 200 | 87 |
| Hammond | 1987 | 59 | 5 | 4 | 2 | 1 | 12 | 61 | 15 |
| Dundas | 1988 | 18 | 21 | 2 | 2 | 0 | 1 | 72 | 32 |
| Smith | 1988 | 110 | 22 | 8 | 12 | 0 | 16 | 307 | 119 |
| Palombini | 1988 | 446 | 24 | 15 | 7 | 0 | 17 | 151 | 10 |
| Langmuir | 1989 | 13 | 15 | 1 | 3 | 0 | 25 | 167 | 33 |
| Wilkinson | 1989 | 29 | 13 | 3 | 0 | 0 | 43 | 21 | 1 |
